# Comparing non-machine learning vs. machine learning methods for Ki67 scoring in gastrointestinal neuroendocrine tumors

**DOI:** 10.1038/s41598-025-08778-6

**Published:** 2025-07-29

**Authors:** Nazanin Mola, Hrafn Weishaupt, Valentin Krasontovitsch, Erlend Hodneland, Sabine Leh

**Affiliations:** 1 Department of Pathology, Haukeland University Hospital, Post Office Box 1400, 5021 Bergen, Norway; 2 Department of Clinical Medicine, Faculty of Medicine, University of Bergen, Bergen, Norway; 3 Department of Medical Genetics, Haukeland University Hospital, Bergen, Norway; 4 Department of Mathematics, University of Bergen, Bergen, Norway; 5 Department of Research and Development, Haukeland University Hospital, Bergen, Norway; 6 Mohn Medical Imaging and Visualization Centre, Haukeland University Hospital, Bergen, Norway

**Keywords:** Ki67, Neuroendocrine tumor (NET), Image analysis, Immunohistochemistry, Intra-observer agreement, Machine learning, Image processing, Cellular imaging, Medical imaging, Machine learning

## Abstract

The Ki67 score is a crucial prognostic biomarker for neuroendocrine tumors, but its manual assessment is labor-intensive, requiring the counting of 500-2,000 cells in hotspots. Digital image analysis could streamline this process, yet few comprehensive comparisons exist between different tools. We compared a non-machine learning (non-ML) tool (ImageScope, Leica Biosystems) with a machine learning (ML) tool (Aiforia Create, Aiforia Technologies) on Ki67-stained slides from 10 low proliferative neuroendocrine tumor cases (Ki67 score < 5%, eight regions per slide). Performance metrics based on the coordinates of detected cells were used to assess the capability of image analysis tools to detect (i) total and (ii) Ki67 positive tumor cells, and consequently calculate the (iii) Ki67 score. Manual scoring by an experienced pathologist was used as the reference standard. The ML compared to the non-ML tool showed better performance metrics (F-score 0.90 vs. 0.74) in detecting the tumor cells. Also, the ML tool had a higher agreement with the reference standard in detecting tumor cells (ICC 0.91 vs. 0.62), Ki67 positive tumor cells (ICC 0.70 vs. 0.24), and the Ki67 score (ICC 0.86 vs. 0.45). Our findings highlight the enhanced accuracy of ML-based image analysis in detecting the correct tumor cells, outperforming traditional methods.

**Supplementary Information:**

The online version contains supplementary material available at 10.1038/s41598-025-08778-6.

## Introduction

Neuroendocrine neoplasms are a rare and heterogeneous group of neoplasms originating from neuroendocrine cells. These neoplasms often show a characteristic morphology and express general markers of neuroendocrine differentiation, such as synaptophysin and chromogranin. Biological behavior and response to therapy are dependent on the site of origin, grade of differentiation, and proliferation rate. In particular, proliferation is a critical factor for the prognosis of well-differentiated neuroendocrine neoplasms originating from the digestive tract, known as neuroendocrine tumors (NETs), as differentiation alone does not provide prognostic information^1^. Consequently, the WHO’s classification of NETs in the digestive tract is based on the proliferation rate.

NETs are divided into three grades: G1 or low grade (mitoses < 2 in 2 mm^2^or Ki67 score < 3% [0–2.99%]); G2 or intermediate grade (mitoses of 2 to 20 in 2 mm^2^or Ki67 score 3–20%); and G3 or high grade (mitoses of > 20 in 2 mm^2^or Ki67 score > 20%).

Given the crucial role of accurate tumor grading in prognosis and treatment planning, particularly for distinguishing G1 from G2 NETs that have distinct clinical outcomes, it is essential to rely on robust markers for grading. Proliferation itself can be measured by the mitosis count or Ki67 score, where the latter proved to be a better indicator of biological behavior^2,3^. The reliability of the Ki67 score increases with the size of the evaluated area, as a larger area provides a greater number of positively and negatively stained cells, resulting in a more statistically accurate reflection of the proliferation rate in the whole slide^4^. However, manual cell counting is a time-consuming task, and its application in a large area is impractical in routine diagnostics. As a compromise between time efficiency and reliability of the estimated Ki67 score, counting a minimum of 500-2,000 tumor cells is recommended^1,5,6^. Basing the measurement on a large number of cells is especially crucial for low proliferative tumors, in which counting only a limited number of tumor cells can easily lead to incorrect results^7,8^. Despite this, due to increased workload^9^ pathologists often opt for eyeballing, which in turn suffers from lack of reproducibility, precision, and accuracy and is further affected by inter-observer variability^10,11^. Furthermore, a correct estimation of the Ki67 score relies on familiarity with histopathological subtleties of a given tumor. For instance, the presence of intra-tumoral lymphocytes or heavily inflamed tumor stroma may result in overestimation of the Ki67 score^12^. Therefore, there is a need for an unbiased, fast, and reliable analytical method for Ki67 score estimation.

The advent of digital pathology has enabled a wide variety of computational methods to extract data from digitized tissue slides^13–15^. Digital image analysis can be a reproducible^16^ and faster alternative to manual scoring of Ki67. Several studies have reported on the automated estimation of the Ki67 score in different malignancies, like breast^17^lung^18^colorectal carcinoma^18^and NETs^19^. However, the application of digital pathology still faces challenges, such as (i) distinguishing tumor from non-tumor cells, (ii) proper cell segmentation (dealing with overlapping tumor cells and inhomogeneous staining), and (iii) discerning Ki67 positive from negative tumor cells (with respect to choosing a threshold for positivity).

While several studies have already investigated possible solutions for the above-mentioned challenges^19–21^ there is a limited number of comprehensive efforts comparing the performance between different tools^22^. Furthermore, existing investigations do not assess performance at the individual cell level. For instance, one study^23^ has performed pixel-level evaluation of AI predictions or correlated the total number of detected nuclei with manual counts, without verifying whether the same individual cells were correctly identified. This gap is critical because simply relying on the final number of detected cells or the calculated Ki67 score can lead to overlooking inaccuracies in identifying the correct cells. Assessing performance at the cell level ensures that the image analysis tools detect the correct cells, rather than achieving seemingly accurate counts despite incorrect detections.

The contribution of the current study is to address this specific gap by evaluating two image analysis tools: a commercially available non-machine learning (non-ML)-based image analysis software and a ML-based image analysis platform for calculating the Ki67 score in NETs. The performance of the employed tools was assessed by comparing not only the calculated Ki67 scores and the number of detected (total and Ki67 positive) tumor cells, but also by measuring the spatial distance between the tumor cells identified by the image analysis tools and those annotated by a gastrointestinal pathologist (SL) aiming to determine whether the correct cells were identified.

## Materials and methods

### Biopsies

Biopsy cases (*n* = 39) from gastrointestinal neuroendocrine tumors were collected from the year 2015 to 2017, from the archives of the Department of Pathology at Haukeland University Hospital, Bergen, Norway. The cases consisted of both endoscopic biopsies and resection specimens. To enable a thorough comparison of the performance of the image analysis tools, a broad variety of NETs in terms of location, stromal content and inflammation were chosen. All cases were reported to have a low Ki67 score of less than 5% (Table 1). The original diagnostic slides (Superfrost plus glass) were used, with the biopsy sections having been cut at a thickness of 5 microns (µm) and stained with Ki67 (Clone MIB-1, Dako, M7240) on the Roche Ventana Benchmark (Roche Diagnostics, Rotkreuz, Switzerland). The glass slides were scanned with a Hamamatsu Nanozoomer XR (Hamamatsu Photonics Norden AB, Kista, Sweden) at 40x magnification, resulting in a resolution of 0.23 μm per pixel. Digital slides were accessed through Aperio eSlide Manager 12 (W10-7018) and viewed in Aperio ImageScope (v12.4.0.7018, Aperio ImageScope, Leica Biosystems, IL, US). All materials were acquired, and all methods were carried out in accordance with relevant guidelines and regulations.

**Table 1:** Grading and localization of the neuroendocrine tumors in the training and test dataset.

Total number of samples		Training	Test
		29	10
Grading	G1	25	8
	G2	4	2
Localization	Small intestine	10	5
	Colon	–	1
	Appendix	14	2
	Rectum	2	1
	Stomach	2	1
	Pancreas	1	-

The grading is that of the original pathology reports. The G2 samples had a Ki67 score of less than 5%.

### Training and test datasets

The cases were randomly split into a training/validation dataset (*n* = 29, male/female = 13/16, mean age = 56.9) and a test dataset (*n* = 10, male/female = 6/4, mean age = 63.3). For the training/validation dataset, the whole slide images (*n* = 29) were uploaded to an ML platform, and different regions representing the normal histological variation of each case were used for training and validation. For testing, eight identically sized regions of interest (ROIs) were selected for each case (*n* = 10) by the first author (MSc in biomedicine) under the supervision of an experienced gastrointestinal pathologist (SL). Each test ROI marked a square region with an edge length of 200 μm in the area with the highest number of positively Ki67-stained tumor cells. Approximately 200 cells were contained within each test ROI. For the test dataset, only the ROIs were uploaded to the ML platform. Ki67 scores were calculated for each ROI by dividing the number of positive tumor cells by the number of tumor cells. The overall score for the given slide was determined by summing all the positive and tumor cell counts across the eight ROIs.

### Manual Ki67 score assessment (reference standard)

**Fig. 1:**
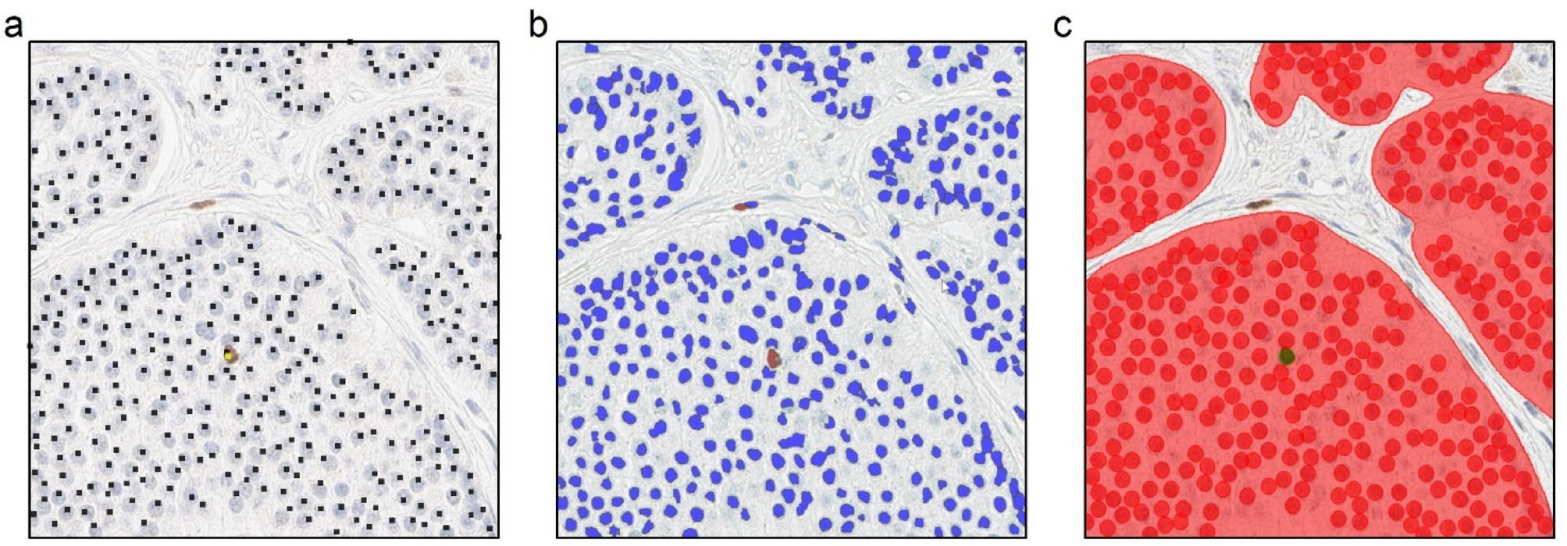
Tumor cell nuclei detection. (**a**) One ROI with the reference standard; black and yellow squares respectively mark all and positive tumor cells. (**b**) Mark-up image resulting from non-machine learning based image analysis with ImageScope; blue and red markings respectively highlight detected Ki67 negative and positive tumor cells. (**c**) Mark-up image resulting from machine learning based image analysis with Aiforia; highlighted red area is the detected tumor region, red and green circles respectively show detected Ki67 negative and positive tumor cells.

The ROIs from the test dataset were exported from ImageScope as .tiff files. Manual annotation was conducted by an experienced gastrointestinal pathologist (SL) on Ki67-stained sections using the dot annotation feature of ImageJ^24^ with the multi-point tool. The results of the annotation process included the number of marked cells on the image and the coordinates of these dot markings. The pathologist (SL) manually counted tumor cells within the ROIs twice, with a wash out period of six months between the counts. Illustrative examples and an instruction to dismiss faintly stained tumor cells were provided for the sake of consistency in the annotations. The intra-observer variability between the two counting rounds was found to be minimal (Supplementary Fig. S1). The second count was considered the reference standard for all comparison with the image analysis tools (Fig. 1a).

### Non-ML based image analysis with Aperio ImageScope

The Nuclear v9 algorithm (Aperio ImageScope)^25^ was used for the automatic detection of total and Ki67 positive tumor cells (nuclei). This algorithm was chosen as it represents a conventional image analysis tool commonly used in research, with a user-friendly interface. Annotations and analyses were performed by the first author (MSc in biomedicine) under the supervision of an experienced gastrointestinal pathologist (SL). Several representative regions in the training/validation dataset were employed to adjust the input parameters to improve the detection and segmentation of tumor cells. The best parameter set (Supplementary Table S1), out of the evaluated configurations, was then applied to analyze the test dataset. Analysis produced mark-up images that classified and color-coded the detected cells on the basis of their staining intensity. Blue and red highlighting were used for negatively and positively Ki67-stained cells, respectively (Fig. 1b).

Since the algorithm did not return any spatial information for the detected cells, we exported the mark-up images into ImageJ and manually annotated the detected cells to obtain their coordinates for later assignment and comparison.

### ML based image analysis with Aiforia

A convolutional neural network was established on Aiforia (Aiforia Technologies Oyj, Helsinki, Finland), a cloud-based ML platform. The use of this ML-based platform was chosen to leverage state-of-the-art technology. It also offers excellent customer support and a user-friendly interface, making it easy to experiment with and implement the ML analysis. For our study, the convolutional neural network consisted of two neural networks. The first neural network segmented tumor areas, and the second network detected Ki67 positive and negative tumor cells within the segmented tumor areas (Fig. 1c). Annotations and analyses, in here also, were performed by the first author (MSc in biomedicine) under the supervision of an experienced gastrointestinal pathologist (SL). The output included the number of detected tumor cells and their center coordinates. The training/validation dataset (*n* = 29) included 6 072 manually annotated tumor areas and 13 030 labeled Ki67 positive/negative tumor cells for training. The AI model was trained from scratch and initialized with random values. The mini batch size was 20 by default and no cross-validation was used. The training dataset was digitally augmented using random scaling of − 20 to 20%, 0 to 10% aspect ratio change, 0 to 10% shear angle, − 20 to 20% brightness and − 10 to 10% contrast change, 0–1% white balance change, and flipping both vertically and horizontally (Supplementary Table S2). The number of training iterations was set to 30,000. The fine-tuning of hyperparameters was conducted in collaboration with Aiforia’s specialists, leveraging their expertise to optimize the model for accuracy and efficiency. The performance of our trained model was evaluated on previously unseen regions from the training/validation dataset and further tested on the independent test dataset, using statistical measures including precision, sensitivity, and the F-score, in addition to visual inspection.

### Cell coordinate transformation

Each image analysis tool uses a different reference system, resulting in the need to transform their output coordinates to facilitate comparability. In particular, the mark-up images generated by ImageScope, which were utilized in ImageJ to manually obtain the coordinates of detected cells, had different resolutions compared to the original ROIs. To address these variations, a python script was developed to transform all the coordinates onto the same grid, by using the size and resolution of the ROIs and mark-up images.

### Cell coordinate assignment

To identify the best match between the transformed coordinates of the cells detected by the image analysis tools and the reference standard, we developed another python script that employed the Hungarian algorithm, which is an optimization method used to efficiently find the optimal one-to-one assignment by minimizing the total distance between the two sets of items being matched^26^.

When comparing the dots representing the coordinates of the reference standard with those of each of the image analysis tools, we observed that there was typically no perfect match. One possible explanation for this disparity is that the pathologist (SL) was not asked to mark the center of the tumor cell nuclei and occasionally placed the dot marking on the corner or edge of the tumor cell. The image analysis tools exhibited similar behavior, for technical reasons. To address the un-matching coordinates, we utilized the maximum diameter (based on visual examination) of a tumor cell as the maximum permitted distance between two sets of coordinates during comparison.

**Fig. 2:**
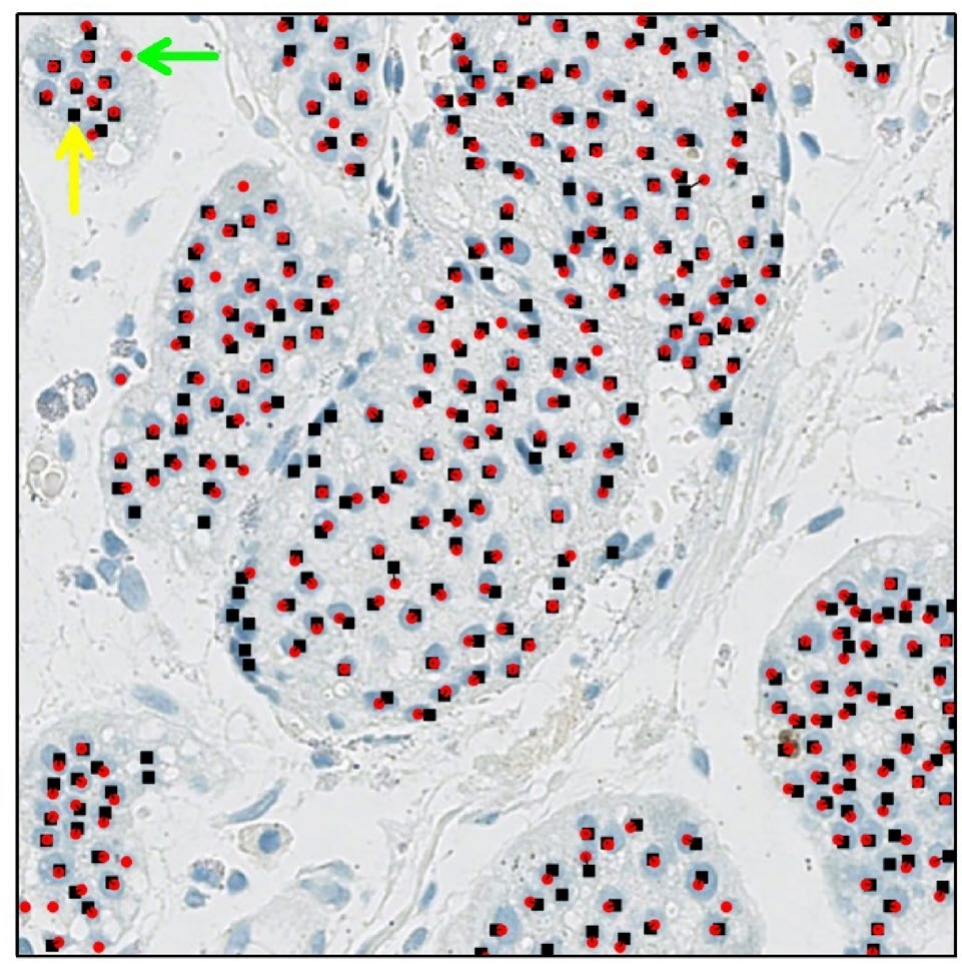
Coordinate assignment for detected tumor cells. Red circles show cells detected by the image analysis tool (in this case, Aiforia), black squares show the reference standard. An isolated red circle (green arrow) represents a false positive (FP) tumor cell detection. An isolated black square (yellow arrow) represents a false negative (FN) tumor cell detection. A successful assignment, a true positive (TP) tumor cell detection, is indicated by a black line connecting the respective matching pair of black square and red circle.

The Hungarian algorithm was used to resolve the multi-to-one assignment cases. For instance, when ImageScope detected several tumor cells in an area where the reference standard identified only one tumor cell, the Hungarian algorithm helped to determine the best matched detections. As a result of the assignment process, matched coordinates were considered true positive (TP) detections, unassigned coordinates from the reference standard were considered false negative (FN) detections, and unassigned coordinates from the respective image analysis tool were considered as false positive (FP) detections (Fig. 2). Furthermore, performance metrics including the false discovery rate (FDR), precision, recall, and F-score were utilized. By utilizing these metrics, in addition to comparing the number of tumor cells detected by the image analysis tools with the reference standard, we were able to evaluate whether each prediction was a correct tumor cell detection.

### Statistical analysis

Statistical analysis was performed using IBM SPSS Statistics (version 26, Armonk, NY, USA, IBM SPSS Statistics). Plots were generated using R (version 4.1.2) within RStudio (version 1.1.463).

The extent of agreement between the results of the image analysis tools and the reference standard was assessed by intra-class correlation coefficient (ICC) with the “two-way mixed” model, based on “single measures” at a 95% confidence interval (CI). The ICC values ranged from 0 (no agreement) to 1 (perfect agreement). A p-value < 0.05 was considered statistically significant^27,28^.

To evaluate the performance of the image analysis tools, metrics such as the F-score, sensitivity, recall, and FDR were used. Total and Ki67 positive tumor cell detection, as well as the Ki67 score were assessed against the reference standard.

For comparing the values of the performance metrics (FDR, Precision, Recall, F-score) between the two employed image analysis tools,, and their number of tumor cell detections, the paired Wilcoxon signed rank test^29^ a non-parametric alternative to the paired t-test, was used. The normal distribution assumption was rejected based on the Shapiro Wilk test.

Bland-Altman plots were employed to visually depict the agreement between the measurements^28,30,31^. Since normality of the data distribution was rejected by the Shapiro Wilk test, a non-parametric method was used. Hence, the limits of agreement were estimated using the 2.5th and 97.5th percentiles and the biases were estimated by the median (instead of the mean) of the differences.

## Results

### Detection of tumor cells

**Fig. 3:**
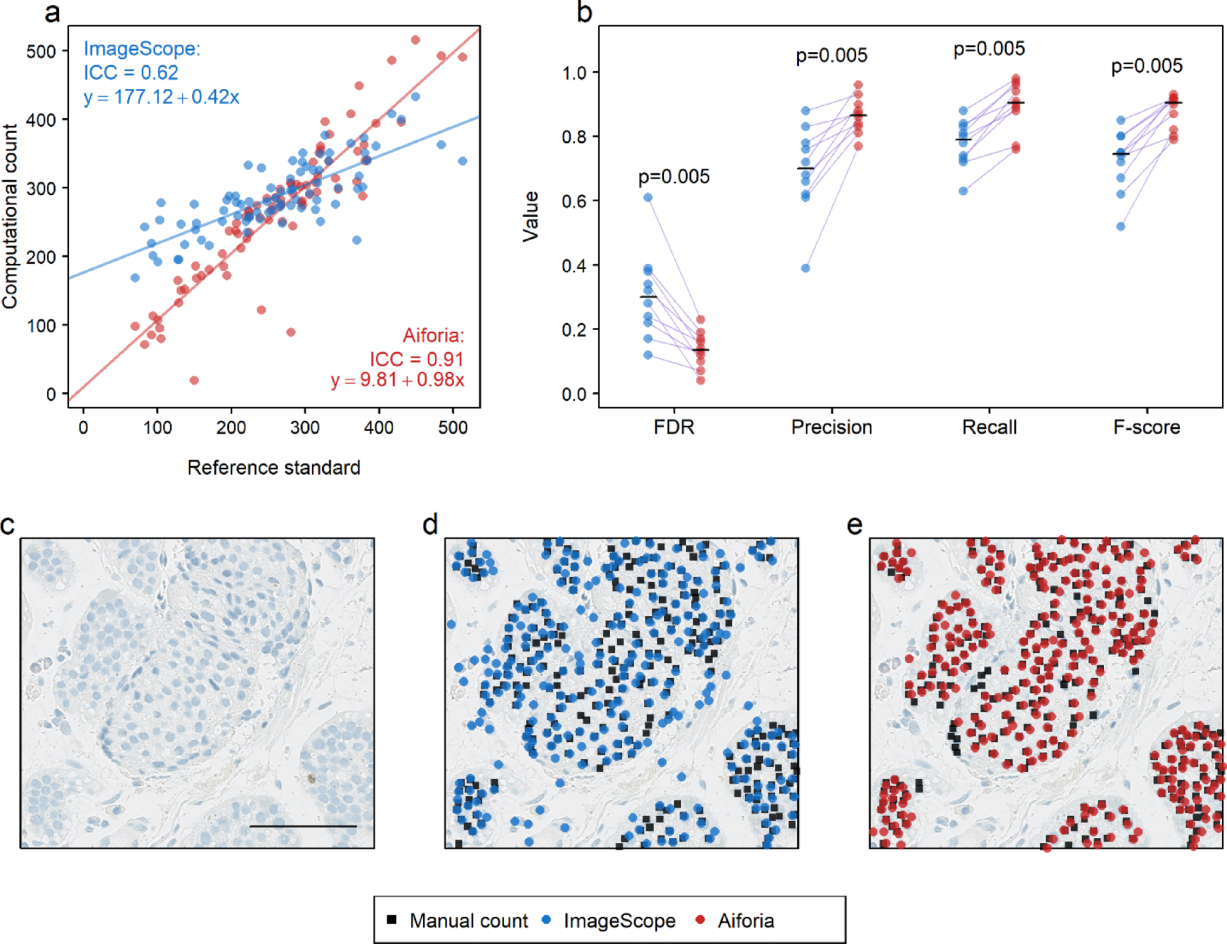
Evaluating the performance of computational detection of all tumor cells. (**a**) Scatterplot and fitted regression lines comparing the total number of detected tumor cells between the reference standard (x-axis) and the image analysis tools (y-axis, ImageScope: blue, Aiforia: red). Each dot indicates the tumor cell count in one of the 80 ROIs in the test dataset. The intra-class correlation coefficient (ICC) represents the extent of agreement between the image analysis tools and the reference standard in detecting the total number of tumor cells. The formula of the regression line between the total number of detected tumor cells by each image analysis and the reference standard is shown. This formula provides insights into the linear association between the two measurements. (**b**) Strip chart comparing a set of performance metrics (false discovery rate (FDR), Precision, Recall, F-score) between the two image analysis tools. Each pair of points are linked by a purple line. *P*-values represent the result of paired Wilcoxon signed rank tests. The median for each set of data points is drawn as a short black line on the datapoints. (**c**–**e**) A single region of interest, the scale bar represents 60 μm. (**d**–**e**) Visualizing total tumor cell detection by the reference standard (black squares) and ImageScope (in **d**, blue circles), and Aiforia (in e, red circles). It is a true positive (TP) detection, for tumor cells, if both the reference standard and the image analysis detection markings are placed on the same cell. A stand-alone black square is a false negative (FN) detection, for tumor cells, and a stand-alone blue (or red) circle is a false positive (FP) detection, for tumor cells.

The evaluation of the two image analysis tools on our test dataset was conducted in three stages. The first stage focused on the detection of total (Ki67 positive and Ki67 negative) tumor cells. When examining the number of detected total tumor cells per ROI, the comparisons of ImageScope and Aiforia against the reference standard revealed moderate and excellent agreement, respectively, with ICC = 0.62 (95% CI, 0.44–0.75) and p-value = 0.0003 (paired Wilcoxon signed rank test) and ICC = 0.91 (95% CI, 0.86–0.94) and p-value = 0.0425 (paired Wilcoxon signed rank test) (Fig. 3a). Out of the 80 ROIs, Aiforia over-counted the total number of tumor cells in 65% (52/80) and under-counted in 35% (28/80), whereas ImageScope over-counted in 70% (56/80) and under-counted in 30% (24/80) (Supplementary Table S3 for detailed counts of tumor cells). While the agreement between Aiforia and the reference standard did not reach the same level as that observed in the intra-observer experiment (ICC = 0.98, 95% CI, 0.97–0.99. Supplementary Fig. S1), the results still indicated that Aiforia performed significantly better than ImageScope did.

Subsequently, we investigated the concordance between image analysis-based tumor cell detection and the reference standard at the level of individual cells, using the four performance metrics (FDR, precision, recall, and F-score). All four metrics were significantly better for Aiforia as compared to ImageScope (Fig. 3b, Supplementary Table S3).

A visual inspection of the prediction results in the ROIs confirmed that ImageScope often misclassified many cells as tumor cells, resulting in FP detection (Fig. 3c, d). Additionally, ImageScope sometimes misidentified overlapping tumor cells as one large cell, leading to FN detection (Supplementary Fig. S2, Supplementary Table S3 for detailed counts of tumor cells). In some cases, the Aiforia algorithm appeared slightly conservative, causing it to miss some true tumor cells, resulting in FN detection (Fig. 3c, e).

In summary, when evaluated for all tumor cells, Aiforia (FDR = 0.13, precision = 0.87, recall = 0.90, F-score = 0.90) presented substantially better agreement with the reference standard as compared to ImageScope (FDR = 0.30, precision = 0.70, recall = 0.79, F-score = 0.74).

### Detection of Ki67 positive tumor cells

In the second step of the evaluation, we examined the detection of Ki67 positive tumor cells in each of the 80 ROIs. The agreement of ImageScope and Aiforia with the reference standard was poor and moderate, respectively, with ICC = 0.24 (95% CI, 0.005–0.44) and ICC = 0.70 (95% CI, 0.46–0.82). Out of the 80 ROIs, Aiforia over-counted the number of Ki67 positive tumor cells in 51% (41/80), under-counted in 6% (5/80), and got matching results in 43% (34/80), whereas ImageScope over-counted in 70% (56/80) and obtained matching results with the reference standard in 30% (24/80) of the ROIs (Supplementary Table S4).

**Fig. 4:**
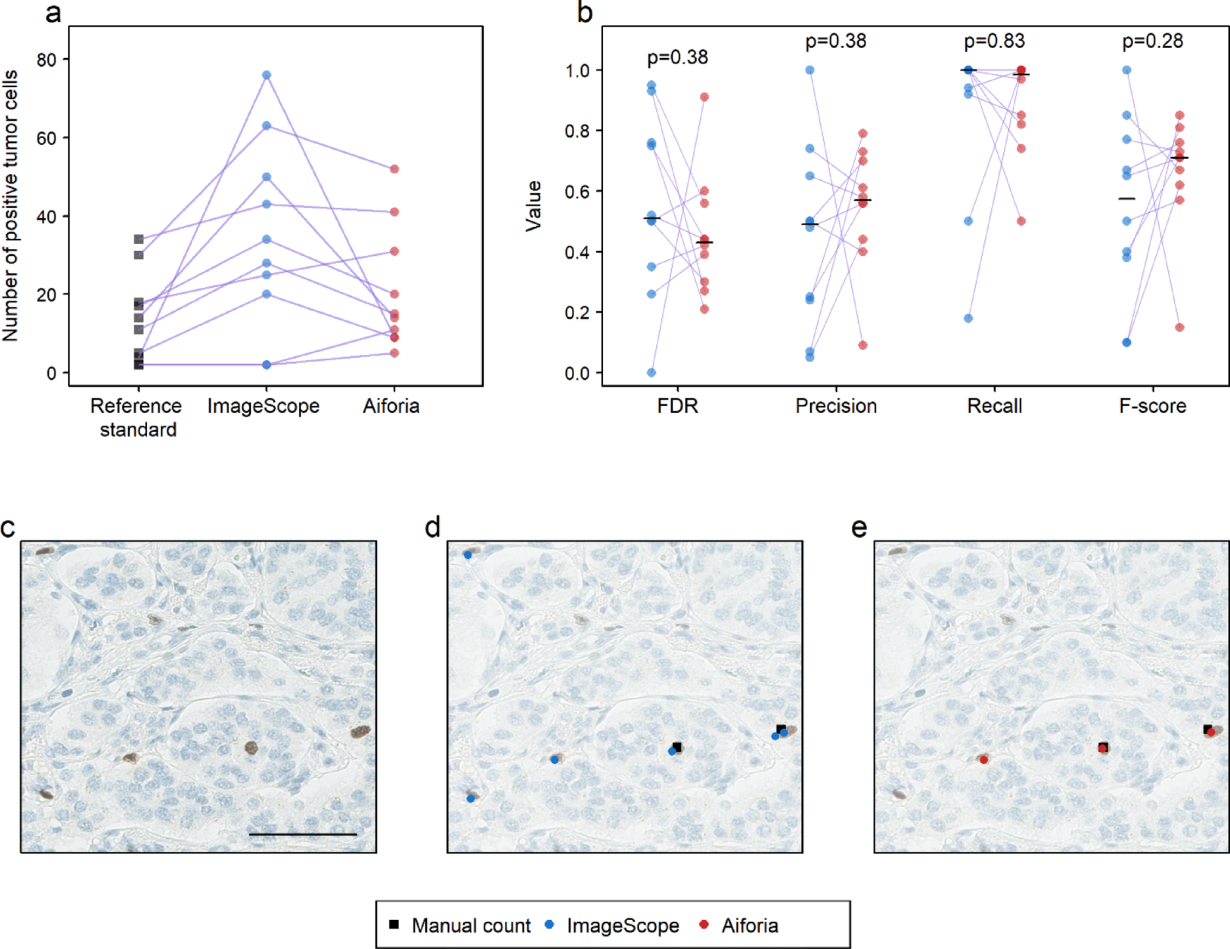
Evaluating the performance of computational detection of Ki67 positive tumor cells. (**a**) Scatterplot showing the distribution of the detected Ki67 positive tumor cells by the reference standard (black squares), ImageScope (blue circles), and Aiforia (red circles), each set of points (linked by a purple line) shows the result across the 10 cases, where an individual point indicates the sum of Ki67 positive tumor cell counts in the eight ROIs for the respective case. (**b**) Strip chart comparing a set of performance metrics (false discovery rate (FDR), precision, recall, F-score) between the two image analysis tools. Each pair of points (linked by a purple line) represents the sum of Ki67 positive tumor cell counts for one of the 10 cases with the respective tool (ImageScope: blue, Aiforia: red). *P*-values represent the results of paired Wilcoxon signed rank tests. The median for each set of data points is drawn as a short black line on the datapoints. (**c**–**e**) A single region of interest, the scale bar is 60 μm. (**d**–**e**) Visualization of Ki67 positive tumor cell detection by reference standard (black squares), ImageScope (in** d**, with blue circles), and Aiforia (in** e**, with red circles). It is a true positive (TP) detection, of a Ki67 positive tumor cell, if both the reference standard and the image analysis detection markings are placed on the same cell. A stand-alone black square is a false negative (FN) detection, for a Ki67 positive tumor cell, and a stand-alone blue (or red) circle is a false positive (FP) detection, for a Ki67 positive tumor cell.

Compared with both the reference standard and Aiforia, ImageScope generally classified almost all positively Ki67-stained cells as Ki67 positive tumor cells, resulting in a higher count. While Aiforia exhibited a slightly more conservative approach than ImageScope in detecting positively Ki67-stained tumor cells, it still counted more compared to the reference standard (Fig. 4a). Based on the number of detected Ki67 positive tumor cells, four performance metrics were calculated for each case. While Aiforia generally showed better values in three out of the four measures (FDR, precision, and F-score), the differences in performance between Aiforia and ImageScope were not statistically significant (Fig. 4b).

Visual inspection revealed that ImageScope tended to split a cell into several smaller parts, which could be due to inhomogeneous staining throughout the cell (Fig. 4c, d and Supplementary Fig. S3). This splitting of cells was more noticeable in the positively Ki67-stained cells and contributed to the higher count of Ki67 positive tumor cells by ImageScope.

Although Aiforia performed better than ImageScope, it still exhibited some errors. It occasionally misclassified Ki67 positive interstitial (Fig. 4c, e) and other Ki67 positive non-tumor cells as Ki67 positive tumor cells or detected faintly stained Ki67 positive tumor cells that were dismissed by the pathologist (SL), resulting in FP detections (Supplementary Fig. S3).

In summary, when evaluated for Ki67 positive tumor cells, Aiforia (FDR = 0.42, precision = 0.58, recall = 0.88, F-score = 0.70) demonstrated better agreement with the reference standard as compared to ImageScope (FDR = 0.61, precision = 0.39, recall = 0.98, F-score = 0.55). However, the differences in performance between the two image analysis tools were not statistically significant.

### Ki67 score

For the third step of the evaluation, we compared the Ki67 scores obtained from ImageScope and Aiforia with the reference standard. The agreement between ImageScope and the reference standard was poor, with an ICC = 0.45 (95% CI, 0.09–0.66). However, Aiforia showed superior agreement with an ICC = 0.86 (95% CI, 0.73–0.92). Out of the 80 ROIs, Aiforia over-estimated the score in 59% (47/80; in some ROIs the image analysis tool counted the Ki67 positive non-tumor cells, or faintly stained tumor cells as Ki67 positive tumor cells), under-estimated in 21% (17/80; in some ROIs the image analysis tool counted more cells by including several non-tumor cells, while counting the same number of positive tumor cells), and provided concordant results in 20% (16/80; all 16 ROIs had no Ki67 positive cells). Whereas ImageScope over-estimated the score in 76% (61/80), under-estimated in 8% (6/80), and gave concordant results in 16% (13/80; all 13 ROIs had no Ki67 positive tumor cells) of ROIs. Notably, that there was one change in grade (for case number 3) in our test dataset by non-ML analysis, compared with the reference standard. None of the cases analyzed by the ML method changed in grade (Supplementary Table S3 and S4 for individual scores of the cases).

**Fig. 5:**
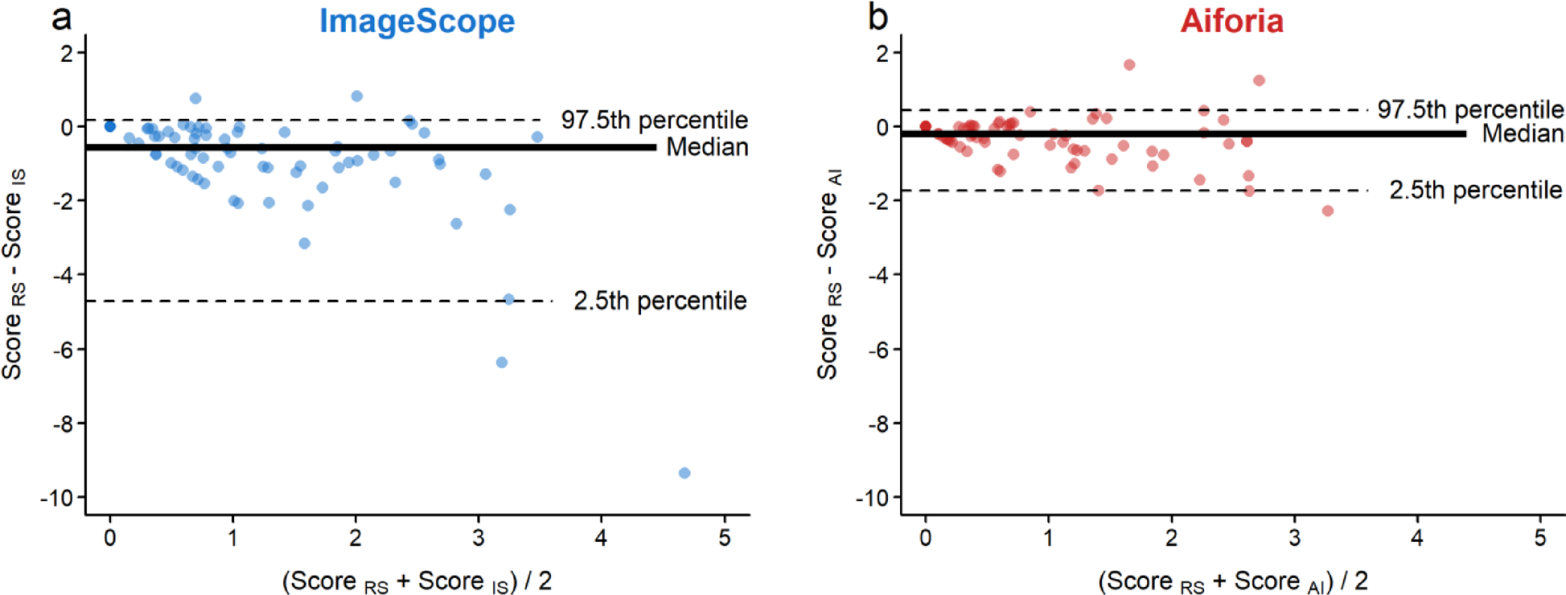
Bland-Altman plot showing the agreement of Ki67 scores between the reference standard (RS) and ImageScope (IS) or Aiforia (AI). The black bold line represents the bias line. The dashed lines are limits of agreement. The x axis shows the average of the score measurements. The y axis shows the difference between the measured scores. (**a**) Agreement between the Ki67 score measured by the reference standard and ImageScope and (**b**) Aiforia for the 80 data points, representing the 80 ROIs in the test dataset. Aiforia has narrower limits of agreements, and its median line is closer to the zero line.

To visually compare the Ki67 scores between the image analysis tools and the reference standard, Bland-Altman plots were created. The plots display the means of the scores on the x axis and the differences in the scores on the y axis for each of the 80 ROIs (Fig. 5) and 10 cases (Supplementary Fig. S4). In both plots, the median line and most data points were below zero, indicating that both image analysis tools over-estimated the Ki67 score compared with the reference standard. Some outliers were observed both below and above the limits of agreement. Aiforia has narrower limits of agreements, and its median line is closer to zero. This finding suggests that when comparing the Ki67 scores with the reference standard, Aiforia demonstrated better agreement and reliability than ImageScope.

## Discussion

Achieving automatic tumor cell detection and Ki67 score estimation is one of the central goals for the application of image analysis in digital pathology. While numerous studies have already addressed the development and evaluation of related image analysis tools^32^ many of these studies focused solely on the number of detected cells or the Ki67 score^33^. However, even if an image analysis tool returns the correct number of detected cells, the same as the reference standard, it does not guarantee that the correct cells have been identified (Supplementary Table S3 and S4 for detailed counts of tumor cells). The current study aims to evaluate detection results at the individual cell level to fully understand any performance differences. To achieve this, performance of a ML-based and a non-ML-based image analysis tool were evaluated in estimating Ki67 score in NETs. The employed non-ML analysis tool represents an example for the conventional approach with a user-friendly interface. While the chosen ML-based platform, supported by strong customer service, exemplifies the state-of-the-art technology and facilitates easy implementation for first-time users. By emphasizing the importance of correct tumor cell identification, our study provides a more comprehensive and clinically relevant evaluation of image analysis tools, ultimately enhancing their trustworthiness and interpretability.

This study specifically focused on challenging NET cases with a low Ki67 score of less than 5%, as accurate estimation of Ki67 score is particularly important for these low proliferating tumors, where a prognostically significant cut-off value of 3% separates G1 from G2 tumors^32^. Misclassifying these cases can have significant therapeutic implication. The WHO recommendation to count 500-2,000 cells is rather vague and does not consider the dependency on the proliferation rate. However, studies in breast cancer have clearly demonstrated that an accurate result depends on both the proliferation rate and the number of cells evaluated^4^. Since manually counting such a large number of cells is impractical, an automated method that can assist in differentiating between G1 and G2 NETs can significantly improve workflow efficiency.

This study demonstrated that neither of the two employed image analysis tools displayed perfect performance after comparing their result at the cell level with the reference standard. However, the ML-based analysis with Aiforia exhibited better agreement with the reference standard in both cell detection and Ki67 score calculation compared to the non-ML-based analysis with ImageScope. Furthermore, it was demonstrated that the improved concordance between the Aiforia’s Ki67 score, and the reference standard was attributed to its ability to distinguish between tumor and non-tumor cells.

The superior performance of the ML-based tool may stem from its use of supervised learning, which enables it to adapt to the data and learn parameters instead of relying on fixed, predefined parameters^34^. However, the performance of Aiforia also showed limitations, manifested by its tendency to pick more positively Ki67-stained cells, including (i) faintly stained Ki67 positive tumor cells which were dismissed by the pathologist (SL), or (ii) Ki67 positively stained proliferating non-tumor cells, resulting in a slightly higher estimated score.

The issue with the detection of faintly stained cells can be attributed to the general difficulty in visually assigning correct labels for weakly stained tumor cells. For those cells, labeling might be highly subjective due to stain quality, section thickness, nucleus appearance, as well as inter-observer and intra-observer variability^35^. The issue of non-tumor cell detection could be due to several underlying reasons, such as limited variation in the training dataset, and narrow control over the training procedures and other hyperparameters, which may limit the ability to fine-tune the model. Vesterinen et al.. reported a mildly better agreement between their trained Aiforia model and manual counting^33^. This could be due to having a bigger dataset. Another possible factor could be differences in the neural network settings. Additionally, they reported only the Ki67 score and did not provide details about their model’s performance in terms of cell counts.

Two main shortcomings in the performance of the non-ML analysis with ImageScope were observed, namely (i) difficulties distinguishing tumor from non-tumor cells, and (ii) issues related to the segmentation of individual cells. Specifically, in the presence of lymphocytes, endothelial cells, and stromal cells, the algorithm would frequently have difficulty distinguishing tumor cells (Supplementary Fig. S5). Depending on the distribution of cell types within a sample, this issue would frequently lead to the over-counting of tumor cells (Supplementary Table S3 and S4 for detailed counts of tumor cells). Non-ML-based analysis tools typically operate with a set of predefined parameters, and thus lack flexibility and adaptability when dealing with complex tasks. One possible solution to address this issue is to limit the scope of analysis, for example to analyze only tumor areas. Using a similar method, Volynskaya et al.. reported that their ImageScope analysis correlated with the pathologists’ results, after manual removal of the stroma within the analyzed regions^36^. While such an approach can improve the analysis results by excluding most non-tumor cells from the analysis, it is more time-consuming and requires domain expertise for precise annotation, making it impractical in our opinion. Other researchers have attempted different strategies, such as (virtual) double staining^32^ or finding complementary biomarkers^37–39^ to facilitate distinction between tumor and non-tumor cells.

The second shortcoming in the performance of ImageScope was related to imprecise segmentations. Two main issues were observed in this regard. First, the method often struggled to accurately distinguish overlapping tumor cells, leading to an underestimation of the number of individual tumor cells (Supplementary Fig. S2). Secondly, ImageScope faces challenges in correctly segmenting Ki67 positive tumor cells in cases of inhomogeneous staining. This issue resulted in overestimation, where one cell was segmented as multiple objects (Supplementary Fig. S3). One approach to overcome the segmentation problem is to measure the area fraction, which compares the area of brown-stained nuclei to the total area of brown- and blue-stained nuclei^40^. However, this solution can be sensitive to tumor type and cell size, making it less universally applicable. While efforts have been made to address this issue^21^ a universally applicable solution has not yet emerged.

Our study stands out as one of the few that specifically investigated the use of ML for estimating the Ki67 score in gastroenteropancreatic NETs. Other similar studies have also developed ML models, either using proprietary software^33^ or open source software^32,41,42^. Using proprietary software, like Aiforia^43,44^ offers certain advantages, such as fast and reliable customer support and often a more intuitive user interface. These factors make it easier, even for pathologists, to fine-tune an ML model. However, there are potential issues associated with proprietary closed source software, including cost, limited insight and control over individual settings and parameters, and ownership of the final trained model. Conducting similar analyses using open source software provides an alternative approach. In this case, more technical knowledge is required to implement and fine-tune the model, but it avoids the issues associated with proprietary software. The choice between proprietary software and open source software depends on various factors, including the specific requirements of the study, available resources, and the technical expertise of the researchers.

Even though Ki67 staining is a routine procedure in most histology laboratories, there can be a substantial amount of staining variation in terms of the brown color of positively Ki67-stained cells. This variation has the potential to affect prediction results across different slides. While the current study takes into account the presence of stain variations when tuning algorithms, additional approaches specifically targeting this issue could involve the use of stain normalization techniques^45^or virtual Ki67 staining methods^46^. An even more effective strategy could be the application of virtual double Ki67/synaptophysin staining, which not only addresses staining variation, but also helps in identifying tumor cells, facilitating a better distinction between tumor and non-tumor cells^47^.

A common challenge in evaluating automated tumor cell detection tools is the lack of an absolute ground truth for comparison. Instead, an approximative ground truth is usually established by a domain expert. In our study, we considered the manual counting of tumor cells by an experienced gastrointestinal pathologist to be a fair representation of the ground truth. The choice of using only one domain expert in setting the reference standard, i.e., not considering the expected inter-observer variability, could be regarded as an additional limitation. However, we believe this approach is reasonable considering the focus of the study, which is comparing the performance of two image analysis tools in detecting the correct tumor cells.

While hotspot selection is often considered a central step in Ki67 scoring, there are no precise instructions on how to choose a hotspot or assess one; for a discussion of this topic see for example Volynskaya et al.^36^. Some studies have explored the reliability of Ki67 scores when averaging several smaller hotspots or using a single larger hotspot^48–51^while others have employed automatic hotspots selection^52–54^. Since the Ki67 score is affected both by the number and size of chosen hotspots, any differences in these parameters between the compared methods could make direct comparison unreliable. In light of these issues, we chose to use the term ROI rather than the term hotspot and decided to define ROIs with a standard size containing approximately 200 cells, which was considered adequate to meet the aim of this study – to compare performance’s accuracy of two image analysis tools in cell level.

In conclusion, accurately determining the Ki67 score is crucial for grading NETs, predicting patient prognosis, and guiding treatment decisions. The integration of automated image analysis tools in clinical practice can enable pathologists to evaluate larger portion of the biopsy in less time. In the current study, we compared two image analysis tools for cell detection and Ki67 score estimation in NETs with low proliferation index and highlighted the importance of evaluating performance at the individual cell level. In comparison with the chosen reference standard, the ML based image analysis demonstrated a better agreement in correctly detecting individual tumor cells, both Ki67 positive and negative, compared to the non-ML approach, resulting in improved Ki67 score calculation.

Our study is among the few publications that compares image analysis tools in a diagnostic setting at the individual cell level. Unfortunately, there is a lack of comprehensive evaluations and comparisons of image analysis tools, particularly in the rapidly expanding market of commercially available artificial intelligence software. The lack of such evaluations hampers the ability of pathology departments to make informed decisions when purchasing these tools. Additionally, existing publications often provide incomplete assessments of model performance, further highlighting the need for more rigorous and comprehensive evaluations.

In the future, we aim to integrate the ML-based image analysis tool in routine diagnostics. A possible integration scenario can be for ML to flag the borderline cases, those near the G1/G2 threshold, for a more comprehensive manual review. This would help reduce the consequences of systemic bias and misclassification by image analysis tools. It can also output confidence scores, enabling pathologists to focus on low confidence predictions. This will involve further validation studies, including detailed analyses of comprehensive datasets of NET subtypes, outcome studies in patients with NETs, as well as the use of large external tumor datasets to validate the performance of the image analysis tool. Clinical validation, which involves correlating the calculated Ki67 score with clinical outcome data, is another important step to ensure the reliability and usefulness of the algorithm in real-world scenarios.

## Electronic supplementary material

Below is the link to the electronic supplementary material.

Supplementary Material 1.

Supplementary Material 2.

## Data Availability

All the source code used to derive the results presented within this study will be made freely available at https://bit.ly/4glwLlA. The test dataset (80 ROIs), which were reviewed and annotated by an experienced pathologist, will be made available at bit.ly/4kg1sub.
