# Supplementary material for: Comparing non-machine learning vs. machine learning methods for Ki67 scoring in gastrointestinal neuroendocrine tumors
Source: Sci Rep. 2025 Jul 29;15:27700. doi: 10.1038/s41598-025-08778-6 (PMC12307702; doi:10.1038/s41598-025-08778-6)
Supplement: Supplementary file 1 — Supplementary Material 1. [file 41598_2025_8778_MOESM1_ESM.pdf]

Supplementary Table S1. Input parameters of the Nuclear algorithm for tumor cell detection in non-ML analysis. This table displays the complete set of parameters used during the analysis. The parameter selection was based on iterative optimization to achieve the most accurate segmentation and quantification of features relevant to the study.

Attribute	Value
Algorithm	Nuclear v9
Algorithm inputs	***Algorithm inputs***
Version	9.2
Image Zoom	1.
Classifier	Nona
Class List	
Classifier Neighborhood	0
Pixel size ()	0.2333
Clear area intensity	240
Counterstain intensity	0.696
Counterstain red OD	0.643
Counterstain green OD	0.317
Counterstain blue OD	0.244
Biomarker 1 red OD	0.509
Biomarker 1 green OD	0.825
Biomarker 1 blue OD	0
Biomarker 2 red OD	0
Biomarker 2 green OD	0
Biomarker 2 blue OD	All stains
Type	Automatic
Method	0
--threshold lower limit	255
--threshold higher limit	1.3
Smoothing ()	6
Smoothing (pixel)	0.65
Merging	High
Trimming	10
Min size ()	184
Min size(pixel)	90000
Max size	1.65307e+006
Max size (pixel)	1.e-002
Roundness	0
Compactness	0.3
Elongation	0
Remove light objects	162
Weak (1+) threshold	160
Moderate (2+) threshold	159
Dark nuclei removal	0
Display plots	No

Supplementary Table S2. The hyperparameters for ML analysis with Aiforia. The hyper parameter fine-tuning strategy was established with the help of Aiforia's customer service.

**Parameters**	**Values**
**Training Process**	
Weight decay	0.0001
Initial learning rate	0.1
Maximum final learning rate	0.0001
Iteration without progress	1000
Mini-batch size	20
Neural network structure version	Default
AI engine version	2
**Region Layer**	
A priori weight	0.5
Desired training window size	8
Training window size (min/max)	256 px _ 2048 px
**Image Augmentation**	
Affine transformation	
Scale (min/max)	-20 _ 20
Aspect ratio	20
Maximum shea	20
**Tonality**	
Luminance (min/max)	-20 _ 20
Contrast (min/max)	-20 _ 20
Maximum white balance change	5
**Image Quality**	
White noise	10
JPG compression quality (min/max)	40 _ 60
JPG compression percentage	0.5
Blur maximum pixels	1
Blur percentage	0.5
**Basic Parameters**	
Field of view	50 µm
Complexity	Extra complex

Supplementary Table S3. Performance measures in tumor cell detection. a) Total number of tumor cells detected by the reference standard (manual counting) and Aiforia. After cell level evaluation, number of correctly detected tumor cells (true positive tumor cell detection, TP), number of non-tumor cells that were detected as tumor cells (false positive tumor cell detection, FP), and number of correct tumor cells that were missed (false negative tumor cell detection, FN) were calculated. The performance metrics of false discover rate (FDR), precision, recall, and F-score were also calculated. **b)** Shows the similar table for ImageScope.

a

**Cases**	**Manual counting**		**Aiforia**								
	**Ki67 score %**	**# Total tumor cells**	**Ki67 score %**	**# Total tumor cells**	**# TP**	**# FP**	**# FN**	**FDR**	**Sensitivity**	**Precision**	**F-score**
Case 1	1.31	2588	1.72	2382	1992	390	596	0.16	0.84	0.77	0.80
Case 2	0.07	2992	0.39	2848	2654	194	338	0.07	0.93	0.89	0.91
Case 3	0.19	2099	0.46	1957	1593	364	506	0.19	0.81	0.76	0.79
Case 4	1.49	873	1.42	986	764	222	109	0.23	0.77	0.88	0.82
Case 5	0.98	1741	1.05	1913	1582	331	159	0.17	0.83	0.91	0.87
Case 6	0.08	2479	0.18	2793	2437	356	42	0.13	0.87	0.98	0.92
Case 7	1.57	1908	2.5	2080	1795	285	113	0.14	0.86	0.94	0.90
Case 8	0.31	1597	0.52	1722	1547	175	50	0.10	0.90	0.97	0.93
Case 9	0.45	2470	0.65	2316	2212	104	258	0.04	0.96	0.90	0.92
Case 10	0.79	2276	1.26	2466	2176	290	100	0.12	0.88	0.96	0.92
Median of performance metrics for Aiforia								0.13	0.87	0.90	0.90

b

**Cases**	**Manual counting**		**ImageScope**								
	**Ki67 score %**	**# Total tumor cells**	**Ki67 score %**	**# Total tumor cells**	**# TP**	**# FP**	**# FN**	**FDR**	**Sensitivity**	**Precision**	**F-score**
Case 1	1.31	2588	1.87	2457	1857	600	731	0.24	0.76	0.72	0.74
Case 2	0.07	2992	0.08	2527	2218	309	774	0.22	0.78	0.83	0.80
Case 3	0.19	2099	3.48	2182	1326	856	773	0.12	0.88	0.74	0.80
Case 4	1.49	873	2.77	1804	701	1103	172	0.39	0.61	0.63	0.62
Case 5	0.98	1741	1.57	2170	1467	703	274	0.61	0.39	0.80	0.52
Case 6	0.08	2479	0.08	2616	2177	439	302	0.32	0.68	0.84	0.75
Case 7	1.57	1908	2.8	2247	1400	847	508	0.17	0.83	0.88	0.85
Case 8	0.31	1597	1.02	1961	1287	674	310	0.38	0.62	0.73	0.67
Case 9	0.45	2470	1.04	2693	1926	767	544	0.34	0.66	0.81	0.72
Case 10	0.79	2276	1.07	2434	1894	540	382	0.28	0.72	0.78	0.75
Median of performance metrics for ImageScope								0.30	0.70	0.79	0.74

Supplementary Table S4. Performance measures in positive tumor cell detection. a) Number of Ki67 positive tumor cells detected by the reference standard (manual counting) and Aiforia. After cell level evaluation, number of correctly detected Ki67 positive tumor cells (true positive detection, TP), number of non-tumor positively Ki67-stained cells that were detected as positive tumor cells (false positive detection, FP), and number of correct Ki67 positive tumor cells that were missed (false negative detection, FN) were calculated. The performance metrics of false discover rate (FDR), precision, recall, and F-score were also calculated. **b)** Shows the similar table for ImageScope.

a

Cases	Manual counting		Aiforia								
	Ki67 score %	# Positive tumor cells	Ki67 score %	# Positive tumor cells	# TP	# FP	# FN	FDR	Sensitivity	Precision	F-score
Case 1	1.31	34	1.72	41	25	16	9	0.39	0.73	0.61	0.66
Case 2	0.07	2	0.39	11	1	10	1	0.91	0.50	0.09	0.15
Case 3	0.19	4	0.46	9	4	5	0	0.56	1.00	0.44	0.62
Case 4	1.49	13	1.42	14	11	3	2	0.21	0.85	0.79	0.81
Case 5	0.98	17	1.05	20	14	6	3	0.30	0.82	0.70	0.76
Case 6	0.08	2	0.18	5	2	3	0	0.60	1.00	0.40	0.57
Case 7	1.57	30	2.5	52	29	23	1	0.44	0.97	0.56	0.71
Case 8	0.31	5	0.52	9	5	4	0	0.44	1.00	0.56	0.71
Case 9	0.45	11	0.65	15	11	4	0	0.27	1.00	0.73	0.85
Case 10	0.79	18	1.26	31	18	13	0	0.42	1.00	0.58	0.73
Median of performance metrics for Aiforia								0.42	0.88	0.58	0.70

b

Cases	Manual counting		ImageScope								
	Ki67 score %	# Positive tumor cells	Ki67 score %	# Positive tumor cells	# TP	# FP	# FN	FDR	Sensitivity	Precision	F-score
Case 1	1.31	34	1.87	46	34	12	0	0.26	0.76	0.74	0.85
Case 2	0.07	2	0.08	2	2	0	0	0.00	0.78	1.00	1.00
Case 3	0.19	4	3.48	76	4	72	0	0.95	0.88	0.05	0.10
Case 4	1.49	13	2.77	50	13	37	0	0.74	0.61	0.26	0.41
Case 5	0.98	17	1.57	34	17	17	0	0.50	0.39	0.50	0.67
Case 6	0.08	2	0.08	2	1	1	1	0.50	0.68	0.50	0.50
Case 7	1.57	30	2.8	63	30	33	0	0.52	0.83	0.48	0.65
Case 8	0.31	5	1.02	20	5	15	0	0.75	0.62	0.25	0.40
Case 9	0.45	11	1.04	28	11	17	0	0.61	0.66	0.39	0.56
Case 10	0.79	18	1.07	26	17	9	1	0.35	0.72	0.65	0.77
Median of performance metrics for ImageScope								0.61	0.98	0.39	0.55
