# Supplementary material for: Comparing non-machine learning vs. machine learning methods for Ki67 scoring in gastrointestinal neuroendocrine tumors
Source: Sci Rep. 2025 Jul 29;15:27700. doi: 10.1038/s41598-025-08778-6 (PMC12307702; doi:10.1038/s41598-025-08778-6)
Supplement: Supplementary file 2 — Supplementary Material 2. [file 41598_2025_8778_MOESM2_ESM.pdf]

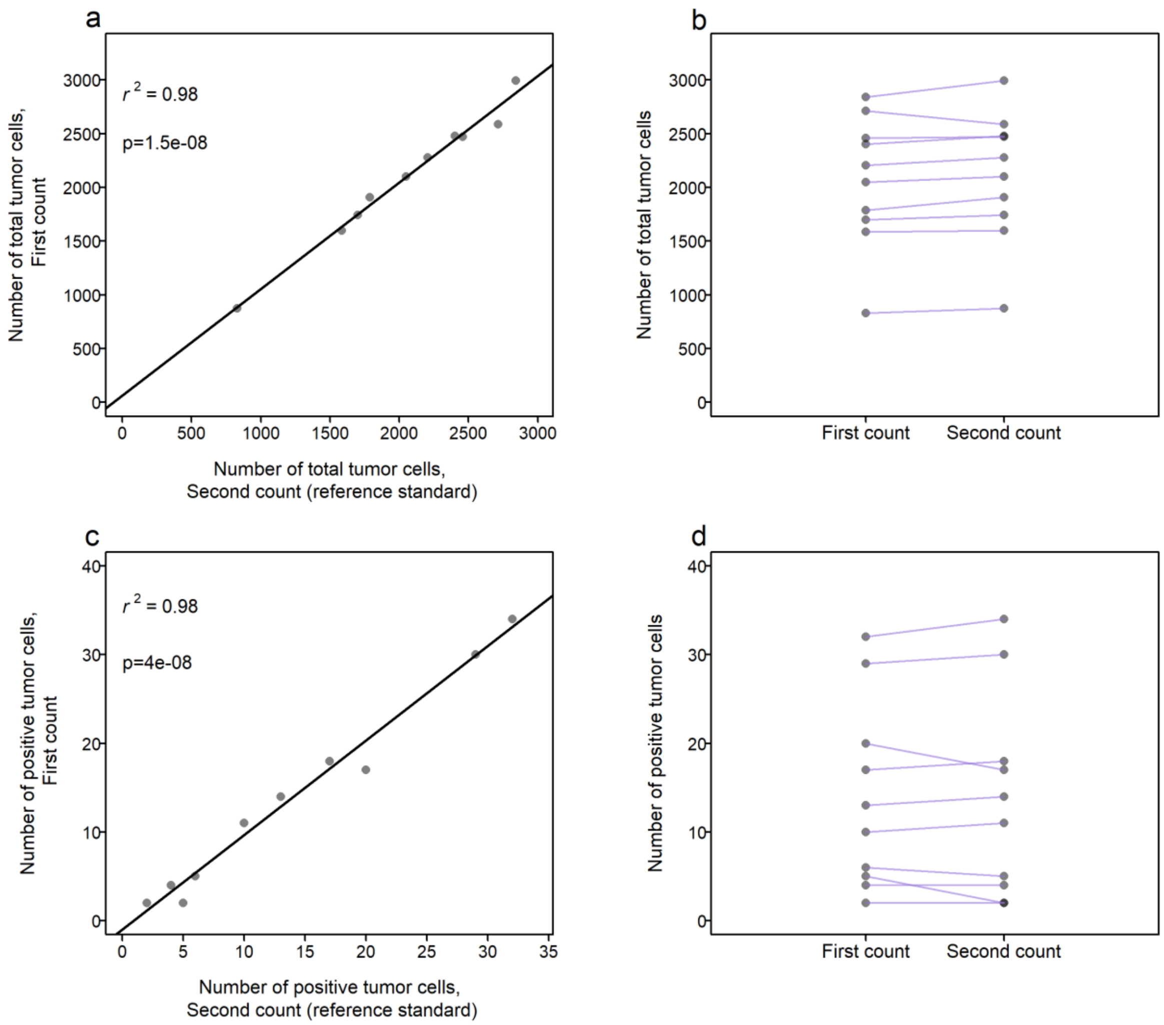

Supplementary Figure S1. Intra−observer variability. The plots show the intra-observer variability between the two counts of total and positive tumor cells, by an experienced pathologist with a wash out period of 6 months. a) Scatter plot showing the correlation between the two manual counts of total tumor cells on the test dataset by an experienced pathologist. Each dot represents the sum of tumor cell counts for one case. R−value refers to Pearson correlation coefficients. P−value represents the result of the paired Wilcoxon rank sum test. b) Stripe chart showing the comparison between the number of detected tumor cells in two rounds of manual counting. Each pair of points is linked by a purple line. c) The same as A, but only for positive tumor cells. d) The same as B, but only for positive tumor cells.

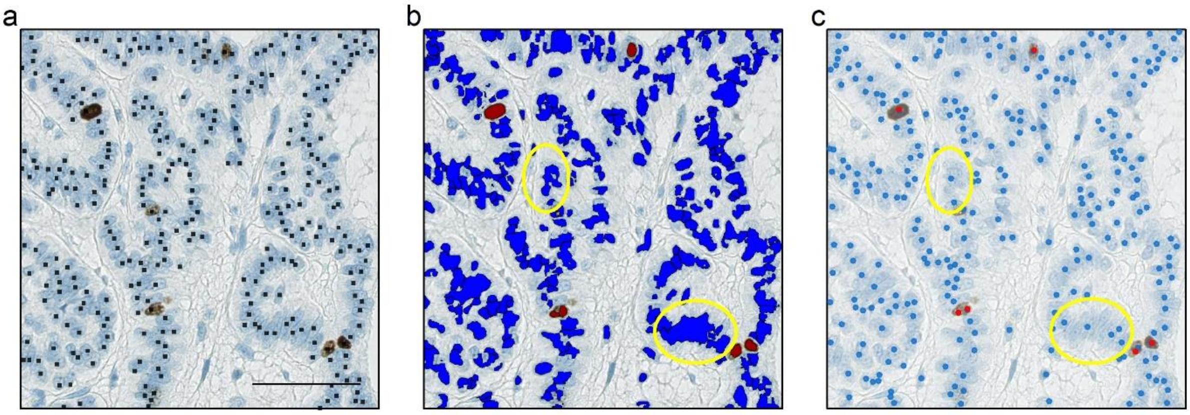

Supplementary Figure S2. Overlapping tumor cells. The figure shows an example of how overlapping tumor cells can lead to under-counting of total tumor cells, resulting in overestimation of the Ki67 score. Overlapping tumor cells make segmentation challenging. A similar effect may also occur in the case of faint tumor cell staining that blurs the edges of tumor cells. A-C: Sample ROI from the test dataset (Ki67 stain, scale bar is 60 µm). a) Reference standard labeling by an experienced pathologist. All tumor cells are marked by black squares. b) Result of the ImageScope analysis shown as mark-up image. Blue and red colors highlight, respectively, the negative and positive tumor cells. Yellow circles indicate two areas with overlapping tumor cells. c) Blue dots highlight detected cells by ImageScope in B. Yellow circles indicate two areas with overlapping tumor cells, showing that the image analysis detected too few tumor cells. The manual count for this ROI resulted in 316 tumor cells, whereas ImageScope detected 282 tumor cells.

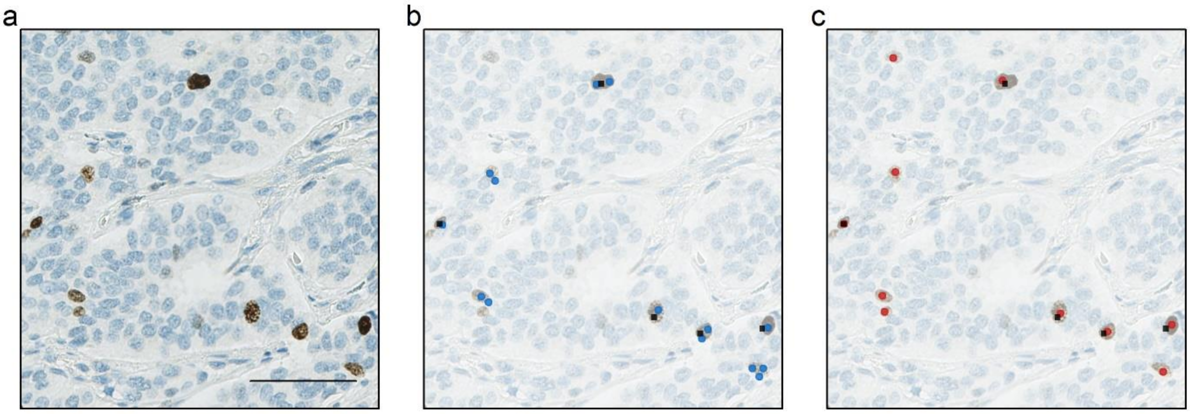

Supplementary Figure S3. One-to-multi detection issue observed with positive tumor cell detection. The figure shows an example of the importance of correct positive tumor cell detection and its impact on Ki67 score calculation. a-c: Sample ROI from the test dataset (Ki67 stain, scale bar is 60 µm). b) Blue circles and black squares, respectively, show ImageScope's positive tumor cell detections and the reference standard. ImageScope detected almost all the positively Ki67 stained cells as positive tumor cells and failed to ignore the faintly stained Ki67 positive tumor cells that were dismissed by the pathologist. The other issue was breaking a positively stained Ki67 cell into several detections, possibly due to inhomogeneous staining throughout the cell. These two issues could lead to overestimation of Ki67 score by ImageScope. c) Red circles and black squares, respectively, show Aiforia's positive tumor cell detection and the reference standard. Here, Aiforia also has difficulty to ignore the faintly stained Ki67 positive tumor cells that were dismissed by the pathologist and hence slightly overestimates the Ki67 score. The reference standard detected 5, Aiforia found 10, and ImageScope reported 14 positive tumor cells.

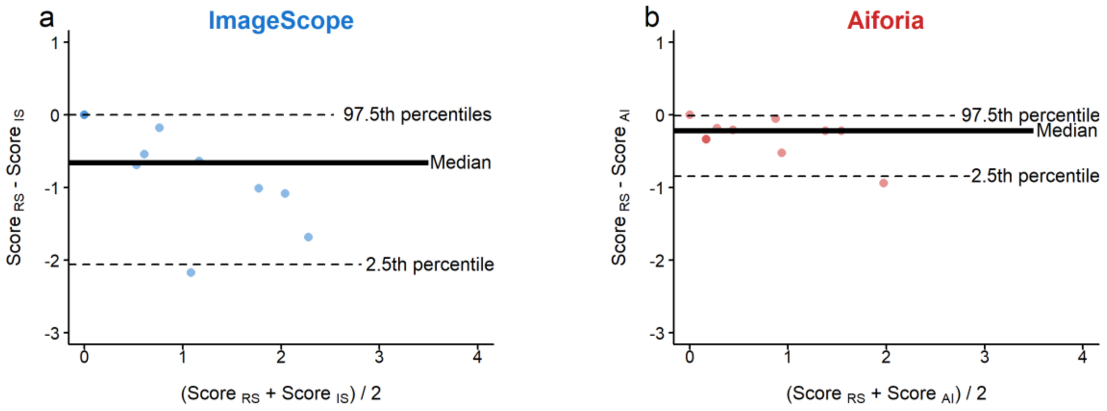

Supplementary Figure S4. Bland-Altman plot showing the agreement of Ki67 scores between the reference standard (RS) and ImageScope (IS) or Aiforia (AI), per case. The black bold line represents the bias line. The dashed lines are the limits of agreement. The x axis shows the average of the score measurements. The y axis shows the difference between the measured scores. Agreement between the Ki67 score measured by reference standard and ImageScope (a) and Aiforia (b) for the 10 cases of the test dataset. Aiforia has narrower limits of agreements, and its median line is closer to the zero line.

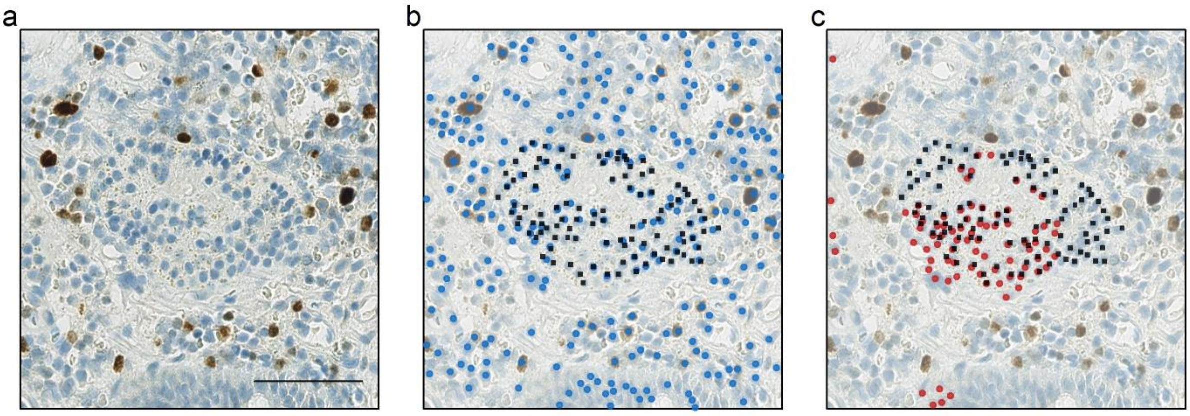

Supplementary Figure S5. Differentiation between tumor vs. non-tumor cells. The figure shows an example of a difficult case and limitations of employed automatic image analysis tools to distinguish tumor from non−tumor cells. a-c: Sample ROI from the test dataset (Ki67 stain, scale bar is 60 µm). b) Blue circles and black squares, respectively, represent ImageScope's tumor cell detections and the reference standard. Here, ImageScope misclassified many non-tumor cells as tumor cells. This can compromise the reliability of the Ki67 score. c) Red circles and black squares, respectively, represents Aiforia's tumor cell detection and the reference standard. Although Aiforia also misclassified a few non-tumor cells as tumor cells and missed several true tumor cells, it generally had a better recognition of tumor and non−tumor cells. The reference standard detected 105, Aiforia found 80, and ImageScope reported 278 tumor cells.
